# Supplementary material for: Identification and functional analysis of non-coding regulatory small RNA FenSr3 in Bacillus amyloliquefaciens LPB-18
Source: PeerJ. 2023 May 15;11:e15236. doi: 10.7717/peerj.15236 (PMC10194069; doi:10.7717/peerj.15236)
Supplement: Supplemental Information 4 [file peerj-11-15236-s004.zip › KO/CK-vs-T1_map/map04011.html]

KEGG PATHWAY: MAPK signaling pathway - yeast - Reference pathway


|  |  |
| --- | --- |
| **MAPK signaling pathway - yeast - Reference pathway** |  |

[
Pathway menu
| Organism menu
| Pathway entry
| Show description
| User data mapping
]

The S. cerevisiae genome encodes multiple MAP kinase orthologs. One (Fus3) mediates cellular response to peptide pheromones. Another (Kss1) permits adjustment to nutrient limiting conditions. A third (Hog1) is necessary for survival under hyperosmotic conditions. A fourth (Slt2/Mpk1) is required for repair of injuries to the cell wall. As in mammalian cells, these pathways consist of a conserved module in which three kinases phosphorylate each other in sequence. The MAPK is phosphorylated by the MAPK/ERK kinase (MAPKK/MEK), which is itself phosphorylated by a MEK kinase (MAPKKK/MEKK).

|  |  |  |
| --- | --- | --- |
| Reference pathway | 184% 150% 122% 100% 82% 67% 55% | 图片下载 |
